# Clinical efficacy and tolerability of an immune-stimulant^*^ constituted by inactivated bacterial bodies in the prophylaxis of infectious episodes of airways: a double blind, placebo-controlled, randomized, multicentre study

**DOI:** 10.1186/2049-6958-9-58

**Published:** 2014-11-19

**Authors:** Stefano Carlone, Michele Minenna, Paride Morlino, Luigi Mosca, Franco Pasqua, Riccardo Pela, Pietro Schino, Alberto Tubaldi, Emmanuele Tupputi, Fernando De Benedetto, the Buccalin Trial Group

**Affiliations:** Pulmonary Department of San Giovanni-Addolorata General Hospital, Respiratory Diseases I, Via dell’Amba Aradan 9, Rome, 00184 Italy; Pneumology, Civile di Bitonto Hospital, Via Gomes 32, Bitonto, BA 70032 Italy; Respiratory Diseases and Respiratory Rehabilitation, Teresa Masselli Mascia Hospital, Via 2 Giugno, San Severo, FG 71016 Italy; Pneumology and Respiratory Physiopathology Department, Hospital of Pescara, Via Fonte Romana 8, Pescara, 65124 Italy; Pneumology Rehabilitation, Villa delle Querce Hospital, Nemi, Rome Italy; Pneumology Unit, C. e G. Mazzoni Hospital, Via degli Iris 1, Ascoli Piceno, 63100 Italy; Department of Pulmonary Disease, F. Miulli Hospital, Strada Prov. 127 Acquaviva - Santeramo Km. 4,100, Acquaviva delle Fonti, BA 70021 Italy; Pneumology Department, General Hospital of Macerata, Via Santa Lucia 2, Macerata, 62100 Italy; Local Health Unit, Hospital District 2, BAT, Via Vittore Carpaccio, Andria, BT 70031 Italy; Pneumology Department, SS Annunziata Hospital, Chieti, Italy

**Keywords:** Bacterial lysates, Immune-stimulation, Placebo-controlled trial, Recurrent respiratory infections

## Abstract

**Background:**

(Buccalin ®) is a Bacterial Lysates (BL) that belongs to a family of immune-stimulators, developed more than 30 years ago and it still has a role in the prophylaxis of Recurrent Respiratory Tract Infections (RRTI). However, original studies were conducted with an approach that does not seem to be aligned with the present methodologies. In addition, concomitant therapies substantially improved in the last decades. These two reasons strongly suggested to update our knowledge on the capacity of this bacterial lysate (Buccalin ®) to reduce the number of days with infectious episodes in patients with RRTI.

**Methods:**

A double blind, placebo-controlled, randomized, multicentre study was programmed (EudraCT code: 2011-005187-25). The reduction of the number of days with infectious episodes (IE) was the primary endpoint. Secondary endpoints were the number of IE, the use of concomitant drugs, the efficacy on signs and symptoms of RRTI and the safety of the drug. Patients were treated according to the registered schedule and were followed up for a period of 6 months.

**Results:**

From a cohort of 188 patients eligible for the study, 90 were included in the active group and 88 in the placebo group. The study was completed in 170 patients. A significant reduction of the number of days with IE was observed (6.57 days in the active group and 7.47 in the placebo group). Secondary endpoints were only partially achieved. No virtual adverse events related to the treatment were recorded.

**Conclusion:**

The administration of bacterial lysate (Buccalin ®) in patients with RRTI had the capacity to significantly reduce the number of days with IE in a multicentre, randomized, placebo controlled, clinical study. The treatment was safe. Of note, all patients were free to be treated with the best concomitant therapies. In these conditions, the positive results observed demonstrated that this bacterial lysate has maintained its capacity of reducing the days with infections in patients with RRTI, also in association to the concomitant therapies available nowadays.

## Background

Recurrent infections of the respiratory tract are frequent not only in children [1], but also in adults [2]. Otitis, rhinitis, sinusitis, pharyngo-tonsillitis, laryngitis, bronchitis and lower airways infections are the most frequent events. In addition, these disease conditions are frequently associated to allergy symptoms, such as rhinitis and asthma. The large use of locoregional and systemic steroid, NSAID and antibiotics, significantly reduces the duration of these diseases, but, at present, few drugs have an impact on the frequency of recurrences as well as the number of days of disease during the cold season [2–5]. Many decades ago, the bacterial lysates were introduced in human therapy in order to control recurrent infections [6]. Notably, it has been shown that patients affected with these diseases are virtually unable to have a naturally induced immune-response at mucosal level [7]. For this reason, different approaches were described to obtain a bacterial lysate suitable to induce an efficient immune-response in treated patients. Thus, the lysis was performed using a chemical approach, such as alkaline lysis, or using physical methods such as heat or mechanical fragmentation. Also the administration route was different, including sublingual administration and gastroenteral absorption. The treatment schedules were different, from few days of treatment per month to a daily administration for months. Finally, also posology was heterogeneous, ranging from a very large number of bacterial bodies-equivalents [8] to lyophilised soluble bacterial proteins [5]. While originally, the clinical effects of these drugs were described and an activation of non specific locoregional immune-response was observed in treated patients [9], the fine mechanism of action of these drugs was clarified only recently. At present, it has been shown that bacteria lysates have the capacity of inducing the maturation of dendritic cells [10, 11] and, following this maturation, a functionally efficient immune-response is expected. Indeed, this initial stimulation of the innate immune system is followed by the recruitment of a functionally efficient recruitment of T and B lymphocytes resulting [12–14] in the secretion of specific IgA directed to administered bacterial antigens in the mucosal fluids, such as saliva [8]. This cooperation between innate and adaptive immune-response was considered responsible for the clinical efficacy of the approach [8, 15].

Of note, these mechanisms were clearly defined for that group of drugs (which included both chemical and mechanical lysates) that are administered in the oral mucosa. Other drugs, such as Buccalin ®, are characterized by a different route of administration. Indeed, Buccalin is constituted by four different microbes (*Streptococcus pneumoniae, Streptococcus agalactiae, Staphylococcus aureus* and *Hemophilus influenzae*) that are killed using heat, then administered as gastro-resistant tablets. Studies that have been carried out on the mechanism of action of bacterial lysate (Buccalin ®) in patients with recurrent respiratory infections demonstrated clinical efficacy of the drug [16–18]. Other studies demonstrated also an increase in the concentration of secretory IgA, suggesting that the administration of this bacterial lysate, either by using the adaptive arm, or by using the innate arm of the immune-response, is efficient in potentiating the locoregional immune-response [19–21]. To this experimental evidence, it should be added that in previous years, the drug has been extensively used to successfully prevent respiratory tract infections. However, it is a common notion that clinical trials, during 80s and 90s, were conducted in a clinical and epidemiological environment different from the present. In particular, the frequency of resistant bacteria, in community acquired respiratory tract infections, was more rare than today and the therapeutic armamentarium available was also different and less powerful. In this context, it should also be noted that the clinical trial rules of those periods were different from the extremely accurate rules of the present.

For all these reasons, a double blind, placebo controlled, multicentre clinical study was conducted to evaluate, using updated and rigorous clinical trial technique, the effect of bacterial lysate (Buccalin ®) on the number of days with IE in patients suffering from RRTI. In this paper, we describe the clinical trial and demonstrate that the treatment with this bacterial lysate is suitable to significantly reduce the number of days with infectious episodes.

## Methods

### Study design

This was a double blind, randomized vs placebo, multicentre clinical study on the efficacy and tolerability of, a bacterial lysate with immune-stimulating properties (Buccalin ®), in the prophylaxis of the infectious episodes of upper and lower airways. Primary endpoint of the study was the reduction of the number of days with infectious episodes in a follow up period of 6 months, starting from the beginning of the treatment, in the group of treated patients, compared with the placebo group. Secondary endpoints were the reduction of a) the number of infectious episodes; b) the frequency and severity of both higher and lower respiratory tract infections, evaluated at 4 and 6 months from the beginning of the treatment; c) the efficacy on different signs and symptoms related to IE. In addition, other secondary endpoints were the disease free period after the end of the treatment, the days of work/school lost and the global efficacy and tolerability, evaluated by the investigators and the well being, evaluated by the patients using a five-point scale. Finally, the occurrence of adverse reactions was also recorded. The study was first approved by the Ethical Committee of the Centre of the Principal Investigator and then by the Ethical Committees of all participants in the study. This study was conducted according to the Good Clinical Practices (GCP) procedures.

### Patient selection

Patients (age 18 – 65) were considered eligible if, during the previous year, a) had suffered from two to six infectious episodes of the respiratory tract, b) were negative for any pathological condition interfering with the present study and c) were able to understand and manage the study protocol. Exclusion criteria were a) the presence of acute (either infectious or non infectious) episodes, requiring hospitalization or intensive therapy at the moment of the randomization; b) gastro-oesophageal reflux; c) auto-immune diseases; d) treatment with immunoglobulins, immune-stimulants, cytokines, interferons, systemic steroids and anti-neoplastic drugs in the two weeks preceding the study; e) known allergy to the study drug; f) pregnancy or breastfeeding; g) other concomitant clinical trial(s); h) incapacity of understanding the protocol because of language or any other reasons.

### Study population

The study was carried out in 10 different centres. Each centre had the objective to recruit 18 patients. Indeed, a total of 180 patients were expected to be enrolled in the study. Ninety were randomized in the active group, whereas 90 in the placebo group. A drop out on 20% was foreseen: for this reason, 140 patients (70 active and 70 placebo) were expected to complete the study and to be evaluable. Indeed, a sample size of 70 in each group was expected to have a 90% power to detect a difference in mean of 10 (Buccalin vs placebo), assuming a common standard deviation of 18, using a two-group t-test with a 0.05 two-sided significance level.

### Randomization protocol

The randomized list was based on the RANUNI random number generator of the SAS software (SAS Institute, Cary, NC, USA). The allocation ratio was 1:1 to treatments bacterial lysate and placebo, with a block size of 4.

### Treatment

The study period was six months and was divided in 4 treatment cycles. Every cycle lasted 30 days: the first 3 days, the treatment (bacterial lysate (Buccalin ®) SIT, gastro-resistant tablets) was administered according to the registration dossier: one tablet on day 1, two tablets on day 2 and four tablets on day 3, while fasting. Buccalin **®** is constituted by a mixture of *Streptococcus pneumoniae* (1 × 10^9^ inactivated bacterial bodies), *Streptococcus haemolyticus* (1 × 10^9^ inactivated bacterial bodies), *Staphylococcus aureus* (1 × 10^9^ inactivated bacterial bodies) and *Hemophilus influenzae* (1.5 × 10^9^ inactivated bacterial bodies). The placebo group received the same schedule but the drug consisted of gastro-resistant tablets containing only excipients (lactose, micro-crystalline cellulose). In the following 28 days patients did not receive any other immune-stimulation. Both the drug and the placebo respectively were produced by SIT srl (AIFA authorization number aM- 229/2009 of December, 11^th^, 2009) and IBNSavio srl (AIFA authorization number aAmm-49/2011 of May, 6^th^, 2011); packaging trials were made by Mipharm srl (AIFA authorization number aM-77/2011 of May 27^th^, 2011) according to the randomization list. Upon request of the investigators, the boxes were sent to the different centres in blocks of 4 blisters. An adhesive part of the label was also stuck to the CRF. Thus, the Clinical Investigators used the unique ID number to identify enrolled patients in the study. Patients, investigators and the biostatistician involved in the study analysis worked in blind conditions. Only after the closure of the database the randomization list was opened.

### Visits

Five visits were scheduled. The first visit started with the evaluation of patients’ eligibility. In eligible patients, the informed consent was obtained, the patient’s history was collected with specific reference to the number and type of infectious episodes and to the treatments adopted. Then, the patient was instructed on the nature of a placebo-controlled study, on treatment schedule and on the compilation of the personal diary. Finally, the patient was randomized, the drug was given and the protocol started. The second visit was scheduled after the end of the fourth week. In this visit, the occurrence of adverse reactions, any day of hospitalization, any day of absence from work and school, the assumption of concomitant therapies (such as NSAIDs, antibiotics, local or systemic corticosteroids, anti cough drugs, mucolytics, expectorants) and any other relevant physiologic or pathologic events were recorded. Then the first month patient’s diary was retired and the compliance to the treatment was verified by retiring the unused study drug. The drug for the following month was given to the patient at the end of the visit. The third and the fourth visit, scheduled after 8 and 12 weeks from the beginning of the treatment, were largely superimposable to the second visit. The fifth and final visit verified all the above-mentioned points. With regard to the sign and symptoms evaluated, the following clinical pictures were considered: otitis, pharyngo-tonsillitis, tonsillitis, sinusitis, rhino-pharyngitis, bronchitis and pneumonia. During visit 1 and 5, the patients were asked to fill in a Visual Analogue Scale (VAS), ranging from 0 (the worst) to 10 (the best situation). At the end of the follow up, the investigators were also asked to declare an estimate of the drug efficacy (in a scale from 0 to 5) and the drug tolerability (in a scale from 0 to 4). Finally, the patients were asked to record the assumption of the study drug. The compliance to the protocol was evaluated at the end of the study, by comparing the number of tables still remaining in the blister and the number of tablets that the patient recorded as assumed.

### Concomitant drugs

The following drugs (immune-stimulants, anti-neoplastic, cytokines, interferons, long term treatment with systemic steroids) were not allowed during the study. Every other treatment was permitted and the patient was asked to record every assumption.

### Definition of acute infectious episodes

Acute episodes of the upper respiratory airways were identified by the continuous presence of rhinorrhoea (both sero-mucous and purulent), pharyngitis, and cough, lasting at least 48 hours, with or without fever. Acute episodes of the lower respiratory airways were defined as the continuous presence of at least one of the following signs or symptoms: stridor, wheeze, crackles and rhonchi indrawing, respiratory frequency >50 cycles/minute, cyanosis, lasting at least 48 hours, with or without fever. Acute episodes of otitis were defined as the continuous presence of pain, erythema and reduced or loss of tympanic membrane mobility. Finally, an acute infectious episode was defined as new if at least 72 hours had passed, in the complete absence of symptoms, from the resolution of the previous episode.

### Patients’ consent withdrawal

Patients were allowed to withdraw from the treatment for any reason. According to the protocol, when possible, the adverse reactions and the use of drugs were recorded in retired patients and the personal diary was retired. Any relevant information was recorded in the CRF.

### Patients’ compliance control

The Investigators instructed the patient to bring the boxes of the drug to each visit. Treatment compliance was calculated at each study visit by the Investigator, by making a cross-check on the vials and using the formula:


The patient was considered as adhering to the therapeutic protocol if he/she had taken at least 80% of the specified quantity of medicine. Treatment compliance was recorded in the CRF by the Investigator.

### Safety assessment

Patients on their patient’s diary and investigators in CRF carefully monitored the occurrence of adverse reactions (AR). These were classified as certain, likely, possible, unlikely, unrelated and not evaluable, according to the definition of the protocol. ARs were also classified as slight (if not interfering with daily activities), intermediate (if interfering with the day activities) and severe (if the AR inhibits daily activities).

### Exclusion of the patient from the study

Patients were excluded from the study because of adverse reaction(s) that, to the investigator’ judgment, were incompatible with the study continuation or if the investigator had the intention to treat the patient with drugs non allowed by the present protocol.

### Patient’s diary

Patients were requested to fill in a diary containing personal information. In particular, in the presence of an infectious episode (IE), a specific diary section was filled in by the patient, indicating a) the beginning and the end of the IE; b) the signs and symptoms (fever, dyspnoea, pain, cough and general status) on a 4 point scale (0 = absent, 1 = mild, 2 = moderate; 3 = severe); c) treatments administered (antibiotics, local or systemic corticosteroids, cough mixtures, mucolytics expectorants, NSAID etc.); d) specialist visits; e) absence from work or school; f) period of hospitalization and g) adverse events.

### Statistical analysis

Statistical analysis was performed on the following three populations: a) Intention-to-Treat (ITT) Population, defined as all randomized patients who received at least one dose of study medication and had at least one evaluation of the primary parameter (assessment of efficacy by the patient’s diary) after randomization. b) Per-Protocol (PP) Population, defined as the set of ITT patients who completed the study without presenting any major violation of the protocol. Major deviations from the protocol were defined in the statistical analysis plan (SAP), approved prior to the opening of the randomization code (breaking the blind). c) Safety or Safety Set (SS) Population, defined as all randomized patients who received at least one dose of study drug. For the evaluation of the efficacy, Intention-to-Treat (ITT) Population was considered as the primary population for analysis. Demographic characteristics and medical history of the patients were summarized for the two study treatments using descriptive statistics (mean, SD, median, for continuous variables and frequency tables for categorical variables). The comparability of treatment groups was assessed by applying appropriate statistical tests, Student's t-test or Wilcoxon Rank-Sum Test for continuous variables, and Chi-Square test for dichotomous variables. The primary variable was the total length, in days, of infections of the respiratory tract observed in the period between randomization and the end of study visit (visit 5, month 6). The results obtained in the two treatment groups were reported using descriptive statistics (mean, SD and median) and compared using the ANCOVA model. The analysis was performed with SAS by using the MIXED Procedure. The statistical analysis and the relevant date listings were produced using the SAS package. The level of statistical significance was fixed at 5% (α = 0.05). All statistical tests were two-tailed. All secondary efficacy variables were evaluated by using mean, SD and median for continuous variables or frequency tables for categorical variables. The comparison between the two treatment groups was made through the application of the Student’s t-test or in the same non-parametric Wilcoxon Rank Sum Test if the parameter did not follow a distribution similar to the normal. The analysis was assessed by applying the Kaplan-Meier method and the comparison between the two groups was evaluated using the log-rank test.

## Results

### Study population

A total of 188 patients (89 females and 79 males) were eligible for the study. Out of these, 90 (49 females and 41 males) were included in the active group and 88 (50 females and 38 males) in the placebo group. One hundred and seventy patients completed the study, 84 in the active group and 86 in the placebo one. Thus, 18 patients (corresponding to less than 20% of the recruited population) completed the study. Out of these, 10 were in the treated group (5 were lost for adverse events, 3 for consensus withdrawal and 2 for protocol violation). The remaining seven patients from the placebo group, were lost for adverse events (two patients), consensus withdrawal (four patients) and the last one was lost during the follow up. Data on FAS were obtained in 178 patients, and data on safety in 181 patients. Smoking habits (84.8% were non smokers and 15.2% smokers) were comparable in the two groups. Similar results were observed for passive smoking (14.6% complained passive smoking). The number of previous infections is shown in Table 1. Of note, there were no differences in relation to gender behaviour.

**Table 1:** **Number of infections in the year preceding the study**

	Placebo	Treated
2	10 (11.4)	11 (12.2)
3	28 (31.8)	22 (24.4)
4	24 (27.3)	20 (22.2)
5	12 (13.6)	18 (20.0)
6	14 (15.9)	19 (21.1)

### Treatment compliance

Patients were particularly compliant to the treatment. In fact, the compliance of the whole group was 99.98%, being that of the active group 100% and that of the placebo group 99.96.

### Primary objective

The primary objective of the study was the demonstration of reduction of the days in which patients suffered from infectious episodes. In the whole period (i.e., from the beginning of the treatment to the end of the follow up, at the end of month 6), the mean number of days with disease in the placebo group was 7.47 (SD 10.61) and in the treated group 6.57 (SD 8.03). ANCOVA analysis on the effect of the treatment resulted F = 4.70, p = 0.032, (significant). The marginal means (i.e. the means that could be expected if the parameters, such as number of previous infections and total number of IE, were balanced), were 7.883 days for the placebo group and 6.159 days for the active group. The length of single infectious episodes (in days) was largely superimposable in the two groups (Wilcoxon p = 0.856). However, it should be noted that 42% of placebo patients and 38% of treated patients had 0 days with infectious disease during the study period. During the follow up period, after the end of the treatment, the number of days with infections was 1.19 in the placebo and 0.48 in the active group (ANOVA p = 0.303, NS). For all these results, no gender differences were observed.

### Secondary endpoints

In details: a) the difference in the total number of infectious episodes was not significant (0.99 and 1.12 for placebo and bacterial lysate, respectively, ANCOVA F = 0.181, p = 0.671, NS). Similar results were obtained when the evaluation was performed at the end of the treatment period (0.86 and 1.04 for placebo and bacterial lysate, ANCOVA F = 0.574, p = 0.450, NS); b) the efficacy on different signs and symptoms related to IE, such as cough, dyspnoea, pain and fever was monitored by using the patients’ diaries and CRF. No significant differences were observed comparing the active group with the placebo one for all these parameters (Table 2). The large difference between the frequency of symptoms in the first four months and the frequency in the last two is partially explained by the fact that the beginning of the study was in late autumn/beginning of winter, when the infections are more frequent than during spring; c) the disease-free period, evaluated as the time of occurrence of the first IE after the end of the treatment cycles, was longer (mean = 57.8 days) in the active group than in the placebo one (mean = 45.8 days), even if not statistically significant (log rank test p = 0.239); e) the use of antibiotics, anti-inflammatory drugs, the association of both and the use of bronchodilators were also evaluated (Table 3). No differences were observed in the use of concomitant therapies, even if the risk of assuming antibiotics was 17% higher (95% CI: 0.63 – 1.17) in the active group, the risk of assuming anti-inflammatory drugs was also 8% (95% CI: 0.503 - 1.681) higher, while the risk of assuming bronchodilators was 11.8% (95% CI 0.367 - 2.119) lower in the treated group. However, none of these statistics were significant; f) the days of work/school lost during the treatment and follow up periods were 1.35 in the placebo group and 1.30 in the active one, ANOVA p = 0.917, NS; g) the well-being, evaluated by the patients using a five point scale showed that no differences were evident, after 4 treatment cycles, between the treated group and the placebo one (7.229 and 7.464 units, respectively, ANOVA p = 0.321, NS). Similar results were observed after 6 treatment cycles; h) the efficacy, evaluated by the investigators using a 6 point scale, showed that only one (1.2%) placebo patient had markedly deteriorated. No differences were observed in 11.9% active and 8.1% placebo patients. An improvement was observed in 56% and 61%, respectively and a marked improvement was observed in 32.1% and 29.1%, respectively. However, these results were not statistically significant (p = 0.584, NS); the tolerability, evaluated by the investigators was excellent in 52.4% and 54.7% of active and placebo patients, good in 46.4% and 43.0%, and moderate in 1.2% and 2.3%, respectively. P was 0.794, NS; finally, k). twenty adverse reactions (related to 18 different patients) were recorded for the active group and 15 (12 patients) for the placebo group. Out of these, one was possibly and two were probably related to the treatment in the active group. In the placebo group, only three were possibly related to the treatment. The treatment suspension was decided by the investigators in four cases, even if in a single patient the relationship with the treatment was considered probable. In two patients treated with the placebo, the study was interrupted and the therapy was suspended, even if any relationship with the administered study was detected.

**Table 2:** **Signs and symptoms evaluated during the clinical trial**

		Months 0-4		Months 4-6	
		Placebo N (%)	Buccalin N (%)	Placebo N (%)	Buccalin N (%)
Cough intensity	Weak	29 (49.2)	42 (57.5)	4 (50.0)	2 (40.0)
	Moderate	26 (44.1)	30 (41.1)	2 (25.0)	2 (40.0)
	Severe	4 (6.8)	1 (1.4)	2 (25.0)	1 (20.0)
	*Total*	*59*	*73*	*8*	*5*
Dyspnoea	Weak	9 (52.9)	19 (70.4)	1 (33.3)	1 (33.3)
	Moderate	6 (35.3)	8 (29.6)	0 (0.0)	1 (33.3)
	Severe	2 (11.8)	0 (0.0)	2 (66.7)	1 (33.3)
	*Total*	*17*	*27*	*3*	*3*
Pain	Weak	16 (69.6)	26 (63.4)	4 (80.0)	4 (80.0)
	Moderate	7 (30.4)	15 (36.6)	0 (0.0)	1 (20.0)
	Severe	0 (0.0)	0 (0.0)	1 (20.0)	0 (0.0)
	*Total*	*23*	*41*	*5*	*5*
Fever	Weak	8 (30.8)	11 (32.4)	1 (25.0)	1 (50.0)
	Moderate	17 (65.4)	23 (67.6)	2 (50.0)	1 (50.0)
	Severe	1 (3.8)	0 (0.0)	1 (25.0)	0 (0.0)
	*Total*	*26*	*34*	*4*	*2*

**Table 3:** **Concomitant therapies**

			Placebo	Bacterial lysate	p
Antibiotics	No	Count	57 (64.8%)	55 (61.1)	
	Yes	Count	31 (35.2)	35 (38.9)	
Anti-inflammatory drugs	No	Count	53 (60.2)	56 (62.2)	0.785
	Yes	Count	35 (39.8)	34 (37.8)	(N.S.)
Antibiotics and anti-inflammatory drugs	No	Count	68 (77.3)	67 (74.4)	0. 659
	Yes	Count	20 (22.7)	23 (25.6)	(N.S.)
Bronchodilators	No	Count	76 (86.4)	79 (87.8)	0. 659
	Yes	Count	12 (13.6)	11 (12.2)	(N.S.)

## Discussion

The administration of bacterial lysates has been suggested in the past for the prophylaxis of infections of both high and low respiratory tracts [3, 16–19]. Since the beginning of this century, a number of clinical and laboratory evidences has been collected to explain the mechanism of action of bacterial lysate and the relevant clinical efficacy of this approach. Briefly, the administration of a bacterial lysate has been shown to induce the maturation of dendritic cells [10, 11], to enlarge the subsets of specific T and B lymphocytes [12–14] as well as to recruit cells of the innate immunity [8–11]. All these involvments of the innate and adaptive immunity produce the secretion of specific antibodies directed to bacterial wall proteins, favouring the opsonisation of living microbes and the subsequent killing mediated by macrophages and granulocytes [7, 8]. For these reasons, the immune system of treated patients seems to be alerted against potential pathogens, resulting in an active prophylaxis of respiratory tract infections.

The composition, the administered dose, the treatment schedule and the route of administration of different bacterial lysates available in human therapy are heterogeneous. Indeed, a bacterial lysate can be constituted by soluble proteins alone, by entire bacterial bodies, by fragmented bacterial bodies and by a mixture of soluble proteins and particulate antigens. The bacterial antigens are administered in mouth, by allowing the adsorption of bacterial fragments or proteins via the oral mucosa, or administered in gastro-protected tablets, capsules or pills, with the aim of allowing bacterial antigens to be absorbed by the gut mucosa. Also the dose and the treatment schedule are heterogeneous, ranging from mg of bacterial bodies to micrograms of proteins, from 10-day cycles to 45 days treatment etc. The bacterial lysate of this study, (Buccalin **®**), is constituted by four different bacterial strains (*Streptococcus pneumoniae, Streptococcus haemolyticus, Staphylococcus aureus and Haemophilus influenzae*) and the total number of bacterial bodies administered in a single tablet is 4.5 × 10^9^. The total dose administered in a cycle is 7 × 4.5 × 10^9^ that is 31.5 × 10^9^ and the total dose administered in the study is 126 × 10^9^. If we compare with other treatments, such as PMBL, the administered dose is 48 × 10^9^ in a day, 480 × 10^9^ in the first ten days of the first month and 1440 × 10^9^ in the first three-month cycle and 2880 × 10^9^ in the two cycles provided. This comparison is harder with other bacterial lysates, such as those containing a soluble fraction of the bacterial antigen.

Despite the great difference between the administered doses, the differences in the clinical outcome are small. Indeed, a significant reduction of the number of days with respiratory tract infections, including otitis, pharyngo-tonsillitis, laryngitis and lung airway diseases, was observed. This reduction was not associated to a reduction of the number of episodes and also was not clearly associated to a reduction of the use of concomitant therapies, such as antibiotics and anti-inflammatory drugs. On the contrary, even if not significant, in patients with severe symptoms, related to cough, fever, pain and dyspnoea, a constant reduction of the number of episodes was observed for patients of the active group.

All together, these results indicate that the treatment with bacterial lysate (Buccalin **®**) was well tolerated, because the number and the severity of adverse reactions was very low, and) a clinical efficacy on the number of days with infectious episodes of the respiratory tract was observed. In addition, patients with more severe symptoms have a trend of more beneficial effects by the treatment. These results have not only a personal impact on patients' health status, but also on the community in which patients are living. Indeed, the reduction of the days with infections not only may be associated to a reduced possibility of the patient to infect relatives, friends and colleagues, but also the economical cost for the community is reduced. Actually, on this topic, the results of this study appear only marginal. Indeed, no clear evidences have been observed on the use of concomitant therapies and on days of absence from school or work. Nevertheless, the number of days with infections was 7.47 in the placebo group and 6.57 in the treated group. The marginal means were even more significant (7.88 and 6.33 for the placebo and the active group), a clear indication that an almost 25% reduction of the number of days with infections, a very good results in this kind of studies, was achieved. This result is in line with those obtained in other clinical studies performed on patients with recurrent respiratory infections in the past, and summarized in review studies [3] and accurate meta-analyses [4].

In this context, strengths of the study are that the observed positive result was achieved in patients that were free to be treated with the best conventional therapeutic armamentarium and that the random design equally distributed the severity of the diseases, the localization of the infections and the concomitant used therapies.

On the contrary, weaknesses of this study are represented by certain heterogeneities of the study population, which included patients with recurrent upper and lower respiratory tract infection, at different degrees of severity and the absence of a specific laboratory support to the follow up.

Other considerations might be kept in mind in order to better understand these results. Actually, the drug was developed many years ago and the dose, the treatment schedule and the composition were based on clinical, experimental and therapeutic data available 20 years ago. Indeed, the concomitant therapies available nowadays are much more effective than those available in the past. To these considerations, it should be added that the number and the severity of respiratory infections are strictly related to the seasonality of the epidemics, the local weather and the characteristics of the winter in which the study was carried out. Even more interestingly, the local climate could strongly impact on the patient's health. All these confounding elements could not be controlled in such a study. For example, all the patients that were eligible, had a number of infections >2 in the previous winter.

## Conclusions

As a matter of fact, about 50% of the same group of patients had no infections during the study. In these conditions, the fact that a significant reduction of the number of days with IE in the treated group was observed, further supports the concept that the treatment with this bacterial lysate (Buccalin **®**) has a positive effect in patients with RRTI.

## Endnote

*(Buccalin ®).

## References

[1] van de Pol AC, van der Gugten AC, van der Ent CK, Schilder AG, Benthem EM, Smit HA, Stellato RK, De Wit NJ, Damoiseaux RA (2013). Referrals for recurrent respiratory tract infections including otitis media in young children. Int J Pediatr Otorhinolaryngol.

[2] Esposito S, Musio A (2013). Immunostimulants and prevention of recurrent respiratory tract infections. J Biol Regul Homeost Agents.

[3] Villa E, Garelli V, Braido F, Melioli G, Canonica GW (2010). May we strengthen the human natural defenses with bacterial lysates?. World Allergy Organ J.

[4] Cazzola M, Anapurapu S, Page CP (2012). Polyvalent mechanical bacterial lysate for the prevention of recurrent respiratory infections: a meta-analysis. Pulm Pharmacol Ther.

[5] De Benedetto F, Sevieri G (2013). Prevention of respiratory tract infections with bacterial lysate OM-85 bronchomunal in children and adults: a state of the art. Multidiscip Resp Med.

[6] Duperret A (1964). Apropos of the treatment of several otorhinolaryngological diseases with Lantigen B aerosols. J Med Bord.

[7] Rossi GA, Peri C, Raynal ME, Defilippi AC, Risso FM, Schenone G, Pallestrini E, Melioli G (2003). Naturally occurring immune response against bacteria commonly involved in upper respiratory tract infections: analysis of the antigen-specific salivary IgA levels. Immunol Lett.

[8] Braido F, Schenone G, Pallestrini E, Reggiardo G, Cangemi G, Canonica GW, Melioli G, Pallestrini E, Reggiardo G, Cangemi G, Canonica GW, Melioli G (2011). The relationship between mucosal immunoresponse and clinical outcome in patients with recurrent upper respiratory tract infections treated with a mechanical bacterial lysate. J Biol Regul Homeost Agents.

[9] Lusuardi M, Capelli A, Carli S, Spada EL, Spinazzi A, Donner CF (1993). Local airways immune modifications induced by oral bacterial extracts in chronic bronchitis. Chest.

[10] Zelle-Rieser C, Ramoner R, Bartsch G, Thurnher M (2001). A clinically approved oral vaccine against pneumotropic bacteria induces the terminal maturation of CD83+ immunostimulatory dendritic cells. Immunol Lett.

[11] Morandi B, Agazzi A, D'Agostino A, Antonini F, Costa G, Sabatini F, Ferlazzo G, Melioli G (2011). A mixture of bacterial mechanical lysates is more efficient than single strain lysate and of bacterial-derived soluble products for the induction of an activating phenotype in human dendritic cells. Immunol Lett.

[12] Lanzilli G, Falchetti R, Tricarico M, Ungheri D, Fuggetta MP (2005). In vitro effects of an immunostimulating bacterial lysate on human lymphocyte function. Int J Immunopathol Pharmacol.

[13] Lanzilli G, Falchetti R, Cottarelli A, Macchi A, Ungheri D, Fuggetta MP (2006). In vivo effect of an immunostimulating bacterial lysate on human B lymphocytes. Int J Immunopathol Pharmacol.

[14] Lanzilli G, Traggiai E, Braido F, Garelli V, Folli C, Chiappori A, Riccio AM, Bazurro G, Agazzi A, Magnani A, Canonica GW, Melioli G (2013). Administration of a polyvalent mechanical bacterial lysate to elderly patients with COPD: effects on circulating T, B and NK cells. Immunol Lett.

[15] Ricci R, Palmero C, Bazurro G, Riccio AM, Garelli V, Di Marco E, Cirillo C, Braido F, Canonica GW, Melioli G (2014). The administration of a polyvalent mechanical bacterial lysate in elderly patients with COPD results in serological signs of an efficient immune response associated with a reduced number of acute episodes. Pulm Pharmacol Ther.

[16] Scotti L, Biondelli G, Borzani M (1987). Use of a polyvalent oral bacterial vaccine in recurrent respiratory infections in children. Minerva Pediatr.

[17] Guerra E, Papetti C, Rosso R, Visconti E, Aiuti F (1992). Utilità di un’associazione di immunoglobuline e vaccino antibatterico polivalente orale nelle infezioni ricorrenti respiratorie. Clin Ter.

[18] Guerra E, Papetti C, Rosso R, Visconti E, Aiuti F (1992). The clinical and immunological efficacy of a combination of immunoglobulins and an oral polyvalent antibacterial vaccine in recurrent respiratory infections. Clin Ter.

[19] Iannello D, Bonina L, Delfino D, Berlinghieri MC, Gismondo MR, Mastroeni P (1984). Effect of oral administration of a variety of bacteria on depressed macrophage functions in tumour-bearing rats. Ann Immunol (Paris).

[20] Bonina L, Arena A, Liberto MC, Iannello D, Merendino RA, Costa GB, Mastroeni P (1988). Impairment of macrophage antiviral activity by soluble tumor products. Effects of bacterial immunomodulators. G Batteriol Virol Immunol.

[21] Fattorossi A, Biselli R, Casciaro A, Trinchieri V, De Simone C (1992). Oral polyvalent vaccine (Buccalin Berna) administration activates selected T-cell subsets and regulates the expression of polymorphonuclear leukocyte membrane molecules. J Clin Lab Immunol.

